# Understanding psychology students’ perspective on video psychotherapy and their intention to offer it after graduation: a mixed-methods study

**DOI:** 10.3389/fpsyg.2023.1234167

**Published:** 2023-10-19

**Authors:** Jennifer Virginie Meier, Josephine Alexandra Noel, Kai Kaspar

**Affiliations:** Department of Psychology, University of Cologne, Cologne, Germany

**Keywords:** behavioral intention, digitalization, learning opportunities, mixed-methods study, student perception, video psychotherapy

## Abstract

**Introduction:**

Video psychotherapy (VPT) demonstrated strong clinical efficacy in the past, with patients and psychotherapists expressing satisfaction with its outcomes. Despite this, VPT only gained full recognition from the German healthcare system during the COVID-19 pandemic. As society increasingly relies on new media, it seems likely that VPT will become even more relevant. Previous studies surveyed practicing psychotherapists and patients about advantages and disadvantages of VPT. In contrast, our approach targets a younger generation, specifically psychology students intending to become licensed practitioners after graduation.

**Methods:**

Our mixed-methods study was conducted in an online survey format and had two main objectives. Firstly, we investigated which person-related variables are associated with psychology students’ behavioral intention to offer VPT after graduation, using a multiple regression analysis. Secondly, we explored psychology students’ perception of advantages and disadvantages of VPT and identified their desired learning opportunities regarding VPT in their study program, using qualitative content analysis.

**Results:**

A sample of 255 psychology students participated. The multiple regression model explains 73% of inter-individual variance in the intention to offer VPT, with attitudes toward VPT showing the strongest relationship with intention to offer VPT. Expected usefulness, satisfaction with video conferencing, and subjective norm also showed significant relations. The students provided 2,314 statements about advantages, disadvantages, and desired learning opportunities, which we coded by means of three category systems. In terms of advantages, the most frequently mentioned categories were low inhibition threshold, flexibility in terms of location, and no need to travel. For disadvantages, the predominant categories included lack of closeness between patient and psychotherapist, lack of nonverbal cues, and problems with technology or internet connection. Regarding desired learning opportunities, training for technical skills, practical application through role-playing and self-experience, and general information about VPT were the most mentioned categories. In addition, we identified numerous other aspects related to these topics, reflecting a differentiated and balanced assessment of VPT.

**Discussion:**

We discuss the theoretical and practical implications of our findings for training the next generation of psychotherapists and outline a specific five-step plan for integrating VPT into study programs.

## 1. Introduction

In recent decades, new technologies for psychotherapy have been developed and used. One of the latest technologies is video psychotherapy. Regarding previous research, there is not yet a consensus on a term describing psychotherapy via video. For example, it was called “psychological therapy via video” (Buckman et al., 2021), “tele mental health conducted via videoconferencing” (Connolly et al., 2020), or “teletherapy” (Giovanetti et al., 2022). The latter sometimes summarizes both psychotherapy via video and via telephone (as in Giovanetti et al., 2022). In this study, we use the term “video psychotherapy” (shortened with VPT), defined as psychotherapy by videoconference that allows psychotherapeutic care in real time and that builds a communication setting in which psychotherapist and patient can see and hear each other by camera and microphone without being in the same place.

VPT showed robust clinical efficacy in multiple studies and meta-analyses (Barak et al., 2008; Kessler et al., 2009; Bower et al., 2013; Karyotaki et al., 2017; Carlbring et al., 2018; Morriss et al., 2019; Giovanetti et al., 2022) as well as similar levels in diagnosis, emergency treatment, symptom relief, and therapy-outcome (Backhaus et al., 2012; Hilty et al., 2013; Norwood et al., 2018; Berryhill et al., 2019a,b). Furthermore, patients reported high satisfaction with VPT, describing it as effective and efficient (Kruse et al., 2017), and psychotherapists reported satisfaction with VPT in general (Ruskin et al., 2004; Interian et al., 2017; Lindsay et al., 2017; Mayworm et al., 2019; Buckman et al., 2021). Although VPT appears to be equally effective as standard face-to-face psychotherapy, VPT was given little attention until the start of the COVID-19 pandemic. In Germany, where the present study took place, the billing of VPT via health insurance was not allowed until 2019. From October 2019, therapists were allowed to deliver 20% of their overall treatment via video conferencing (BPtk, 2019). The pandemic forced psychotherapists and patients to engage in VPT at a large scale (Eichenberg et al., 2021; Ghaneirad et al., 2021). Since March 2020, when contact restrictions were established in many countries, a dramatic shift from traditional face-to-face psychotherapy sessions to video-delivered sessions was observable (Buckman et al., 2021). Online forms of psychotherapy became an important component of psychotherapy practice during the pandemic (Konieczny, 2021). As a result, VPT gained high attention in many countries (Zangani et al., 2022) and was also accepted by health insurance companies (KBV, 2022). Importantly, however, VPT will only be established as part of the health care system in the long run if psychotherapists are motivated to offer this technology to their patients. The central question is therefore whether psychotherapists are willing and prepared to offer VPT as far as technical and legal constraints are given. Few studies have addressed this question, and they found ambiguous results. Monthuy-Blanc et al. (2013) examined the intention of health care providers to offer VPT and were able to explain 68% variance with their model with perceived usefulness as the strongest predictor. Conversely, Hoffmann et al. (2020) showed that specialists felt that therapy via video conferencing was sufficient for initial counseling but was not a true alternative to (long-term) psychotherapy via face-to-face. It seems that practicing psychotherapists are somehow divided about this new possibility of therapy.

One hurdle for practicing psychotherapists could be that they need to become familiar with a new technology. Adopting a new technology always takes time, with younger people being faster at it (Czaja et al., 2006). Furthermore, young people use media more often for communication than older generations (Blackwell et al., 2017). A negative relationship between age and the adoption of a new technology is also evident in work contexts (Meyer, 2011). Therefore, it is important to focus on the next generation of psychotherapists, namely psychology students who intend to be licensed practitioners after graduation. This generation has been neglected in research related to VPT. To fill this empirical gap, the present mixed-methods study addressed two objectives:

First, we examined a set of person-related variables that are expected to determine psychology students’ intention to offer VPT when they become licensed psychotherapists. This quantitative analysis, conducted through multiple regression analysis, provides an assessment of which individual characteristics are particularly important in adopting VPT (Section 1.1).

Second, we conducted an exploratory qualitative content analysis to examine psychology students’ perceptions of the advantages and disadvantages of VPT, as well as their desired learning opportunities within their psychology studies (Section 1.2). This analysis involved systematically categorizing and interpreting the qualitative data obtained from participant responses. The insights gained from this analysis are crucial for informing the development of learning content for future psychotherapists, particularly considering ongoing changes in training programs and regulations. For instance, recent revisions to psychology training regulations in Germany by the Bundesrat (Bundesregierung, 2019) have resulted in an enhanced focus on clinical psychology and a more comprehensive understanding of psychotherapy principles and applications (Linden, 2021). Therefore, the findings of our study provide valuable guidance to universities in addressing the specific needs and preferences of psychology students regarding VPT.

### 1.1. The present research model

**Figure 1:**
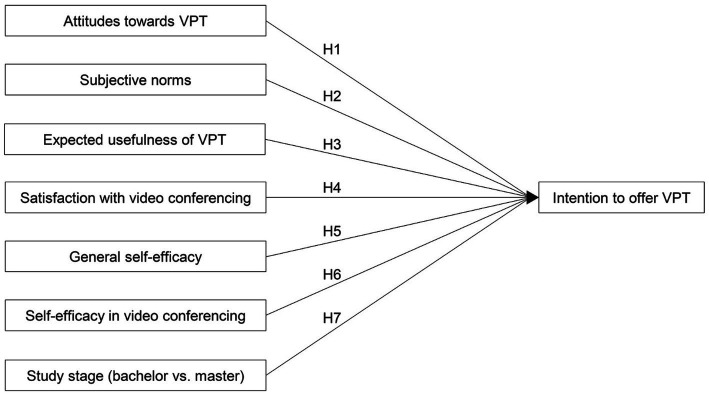
Overview of the research model and corresponding hypotheses of the present study.

In general, the intention to do something is a basic mechanism and prerequisite for performing a behavior (Fishbein and Ajzen, 1975). Therefore, we focused on intention as an indicator of psychology students’ future behavior in terms of offering VPT. Specifically, intention to offer VPT was defined as psychology students’ intention to offer VPT in the treatment of patients (in addition to traditional face-to-face psychotherapy) once they become licensed psychotherapists. Since psychology students have not yet performed treatments themselves and may not have experience with VPT, some variables from known predictive models were not applicable (e.g., perceived behavioral control, ease of use). Furthermore, at the time of the study, it has been unclear how the legal regulations and infrastructure regarding VPT will develop in Germany. These circumstances also explain why we did not investigate actual behavior, but only the intention to offer VPT in relation to person-related variables. In addition to intention as the main dependent variable, our research model incorporated a set of several person-related (independent) variables assumed to explain variance in psychology students’ intention to offer VPT (see Figure 1).

The first model invoked was the Theory of Planned Behavior (Ajzen, 1991), which postulates three conceptually independent determinants of intention: Attitudes toward the behavior, subjective norm, and perceived behavioral control. According to Ajzen (2006), perceived behavioral control represents previous experiences that facilitate or hinder users to perform a behavior. Since the psychology students of the present study had no experience with the performance of VPT, we excluded the variable behavioral control. The second model that we considered was the Technology Acceptance Model by Davis (1989), which postulates three variables affecting the intention to use a technology: attitude, perceived usefulness, and perceived ease of use. According to Davis (1989), perceived ease of use affects perceived usefulness and attitudes toward the technology, but only indirectly affects intention to use. Therefore, perceived ease of use was excluded in our study. Both models were repeatedly used to predict intention of a behavior. Nevertheless, some other variables do not appear in any of the models but showed effects on behavioral intentions in other studies. For this reason, we included four additional person-related variables in our research model: Satisfaction with video conferencing, general self-efficacy, self-efficacy in video conferencing, and current study stage. The following paragraphs provide additional information on the variables included in our research model, as well as the corresponding hypotheses and research questions.

#### 1.1.1. Attitudes toward VPT

According to Greenwald (2014), attitudes are defined as mental and neural representations, influenced by experience, and exerting a dynamic influence on behavior. Attitudes can refer to a person’s state of mind or feelings, whether positive or negative, regarding engaging in the target behavior (Fishbein and Ajzen, 1975; Davis, 1989). Consistent with the Technology Acceptance Model (Davis, 1989) and the Theory of Planned Behavior (Ajzen, 1991), attitudes were defined in this study as positive feelings toward VPT. Davis (1989) stated that the stronger a person’s positive attitude toward technology, the higher their intention to use that technology. Previous research has identified care providers’ attitudes toward online mental health care as the most important factor in intention to use it (e.g., Yarborough and Smith, 2007; Gidenko, 2017). This leads to the following hypothesis:

*H1*: There is a positive relationship between psychology students’ attitudes toward VPT and their intention to offer VPT as licensed psychotherapists.

#### 1.1.2. Subjective norm

In the Theory of Planned Behavior, subjective norm is an important predictor of behavioral intention and it is defined as social pressure to perform or not to perform a behavior (Ajzen, 1991). According to this theory, the degree of subjective norm is higher when an individual perceives that others expect them to perform a behavior (Ajzen, 1991). For instance, Gong et al. (2019) found a positive relationship between subjective norm and the intention to adopt online health consultation services among university students, indicating the influence of subjective norm from the patient’s perspective. Similarly, subjective norm showed a strong relationship with the intention to use technologies among pre-service teachers, whereas a weaker relation was observed among in-service teachers (Ursavaş et al., 2019). Furthermore, in a study inspired by the Theory of Planned Behavior, Hill et al. (1996) concluded from the results of their study that the influence of subjective norm may be especially important for novel types of behaviors. Considering that offering VPT is a novel behavior for psychology students, we formulated the following hypothesis:

*H2*: There is a positive relationship between psychology students’ subjective norm and their intention to offer VPT as licensed psychotherapists.

#### 1.1.3. Expected usefulness of VPT

Perceived usefulness is a key concept in the Technology Acceptance Model (Davis, 1989) and is defined as the degree to which someone believes that the use of a specific technology would enhance their performance (Davis, 1989). Numerous studies showed that perceived usefulness had a strong relation to intention (Davis, 1989; Hu et al., 1999; Venkatesh and Davis, 2000; Chau and Hu, 2001; Monthuy-Blanc et al., 2013; Gidenko, 2017). For example, Monthuy-Blanc et al. (2013) identified expected usefulness as the most crucial factor influencing in-service psychotherapists’ intention to offer VPT to patients. As the psychology students in this study were not yet in-service psychotherapists and had no firsthand experience with VPT, they were asked about the expected usefulness of VPT. Considering the significant positive relationship between perceived usefulness and intention observed in numerous studies, we formulated the following hypothesis:

*H3:* There is a positive relationship between psychology students’ expected usefulness of VPT and their intention to offer VPT as licensed psychotherapists.

#### 1.1.4. Satisfaction with video conferencing

Satisfaction is defined as a global evaluation or a state of feelings toward a service or a product (Olsen et al., 2005). All participants in this study had significant prior experience with video conferencing as it served as the primary mode of remote learning during the COVID-19 pandemic in Germany (*cf.* Hoss et al., 2021, 2022). Additionally, video conferencing software was extensively utilized for maintaining social connections with friends and family during the initial COVID-19 lockdown in Germany (Meier et al., 2021). Shiau and Luo (2013) found that prior experiences can determine user satisfaction with the technology used. Particularly in e-learning contexts, a relationship between satisfaction and intention was found (e.g., Liaw, 2008; Chow and Shi, 2014; Chao, 2019). For example, Chow and Shi (2014) showed that satisfaction with e-learning is positively associated with higher intention to continue using it. Based on this, we expected:

*H4*: There is a positive relationship between psychology students’ satisfaction with video conferencing and their intention to offer VPT as licensed psychotherapists.

#### 1.1.5. General self-efficacy

A person’s ability to overcome barriers and find new solutions in an unfamiliar and difficult environment is conceptualized by Bandura’s (1977) self-efficacy theory. According to this theory, the initiation and maintenance of behavior are influenced by the evaluation and expectation of one’s own abilities, as well as the likelihood of effectively coping with environmental challenges. Sherer et al. (1982) described general self-efficacy as a personality trait that influences an individual’s performance, particularly in unfamiliar situations. Numerous studies used self-efficacy as a variable to assess the influence of individuals’ confidence and willingness to adopt new technologies (*cf.* Rho et al., 2014; Tsai et al., 2019). In their systematic review of technology acceptance, Rahimi et al. (2018) found that the positive effect of self-efficacy has often been investigated in the context of health informatics. For instance, studies showed that individuals with higher levels of self-efficacy were more likely to use online medicine treatments (Viers et al., 2015; Roettl et al., 2016; Vatnøy et al., 2017). Moreover, Wang et al. (2019) showed that self-efficacy, along with enjoyment, were one of two variables in their model that had a significant relationship with the intention to use an e-learning application. This leads to the following hypothesis:

*H5*: There is a positive relationship between psychology students’ general self-efficacy and their intention to offer VPT as licensed psychotherapists.

#### 1.1.6. Self-efficacy in video conferencing

Specific self-efficacy expectations can be deductively derived from general self-efficacy expectations (Schwarzer and Jerusalem, 1999) and should be evaluated within specific domains (Pajares, 1996; Klassen and Chiu, 2010). This relationship has been shown in studies where higher general self-efficacy was related with increased computer self-efficacy and perceived technical competence (Paraskeva et al., 2008; McCoy, 2010). Confidence in video conferencing is necessary for offering VPT. Hence, we included self-efficacy in video conferencing in our model. Consistent with Compeau and Higgins (1995a,b), self-efficacy in video conferencing was defined as the belief in one’s ability to accomplish tasks using video conferencing. The experience and skills related to video conferencing that psychology students already acquired, inter alia via distance learning at university (Marinoni et al., 2020) or video communication with friends and family during the COVID-19 pandemic (Meier et al., 2021), could benefit them when offering VPT in the future. Therefore, we formulated the following hypothesis:

*H6*: There is a positive relationship between psychology students’ self-efficacy in video conferencing and their intention to offer VPT as licensed psychotherapists.

#### 1.1.7. Study stage

According to the Theory of Planned Behavior (Ajzen, 1985), intention can vary depending on the time interval between expressing intention and actual behavior. The longer the temporal gap, the greater the likelihood that unexpected events will lead to changes in intention (Ajzen, 1985). Some changes are likely to occur naturally over time, while others depend on the emergence of new information (Ajzen, 1985). Hence, investigating the differences of psychology students toward offering VPT between bachelor’s and master’s programs is valuable. Master’s students have studied more professionally relevant content and are closer to their future roles as psychotherapists on the other. This resulted in the following non-directed hypothesis:

*H7:* There is a difference in intention to offer VPT as licensed psychotherapists between psychology students enrolled in bachelor’s versus master’s programs.

### 1.2. Explorative qualitative approach

Intention is one of the most important variables for behavioral predictions (Fishbein and Ajzen, 1975) and standardized questionnaires are effective in predicting it. However, when it comes to identifying underlying values, beliefs, and assumptions, qualitative methods are more appropriate (Choy, 2014). Especially in fields where standardized questionnaires are lacking and limited knowledge exists. Qualitative methods offer a broad and open-ended inquiry, allowing participants to address the themes that matter most to them (Yauch and Steudel, 2003). Such exploratory methods can provide valuable insights into new issues emerging in the rapidly changing field of information systems (Choy, 2014). Hence, we complemented the quantitative research approach of our research model with a qualitative study section. We focused on psychology students’ perceptions of the advantages and disadvantages of VPT as well as their desired learning opportunities. The findings from this section can contribute to the preparation of the next generation of psychotherapists in offering VPT.

The following paragraphs summarize the current research on perceived advantages and disadvantages among patients and in-service psychotherapists, as well as on desired learning opportunities.

#### 1.2.1. Perceived advantages and disadvantages

Some studies analyzed perceived advantages and disadvantages of VPT for practicing psychotherapists and patients (e.g., Buckman et al., 2021; Eichenberg et al., 2021). Buckman et al. (2021) aimed to identify the barriers and benefits mental health professionals perceive, which can help to consider how to optimize the delivery of VPT. They identified several perceived benefits, including better work-life balance, efficient use of time, the possibility of recording sessions, and no requirement for a dedicated therapy room. Additionally, in-service psychotherapists described the ability to address difficult issues more quickly, reduced patient shame and inhibitions, and increased focus on the therapeutic process (Eichenberg et al., 2021).

Regarding perceived disadvantages of VPT, in-service psychotherapists reported that during the transition period from face-to-face psychotherapy to VPT due to the pandemic, patients who had not yet established a stable relationship were more likely to discontinue therapy (Eichenberg et al., 2021). Moreover, in-service psychotherapists perceived the lack of nonverbal cues via video as a disadvantage of VPT (Eichenberg et al., 2021). Additionally, they encountered technological issues, experienced increased fatigue after sessions, faced challenges with online platforms, and dealt with internet connectivity problems (Buckman et al., 2021; Eichenberg et al., 2021).

To the best of our knowledge, no previous research has explored the perceived advantages and disadvantages of VPT from the perspective of psychology students. Insights into this perspective can inform students’ concerns regarding VPT and provide valuable knowledge about VPT. Utilizing these insights, the curriculum of the study program can be optimized. For example, discussions on advantages and disadvantages of VPT can be incorporated and further explored in university courses. This could encourage psychology students to feel more confident in offering VPT when they become licensed psychotherapists (*cf.* Aafjes-van Doorn et al., 2021). Therefore, we formulated the following research question:

*RQ1*: Which advantages and disadvantages of VPT are perceived by psychology students, how relevant do they rate advantages and disadvantages in relation to their intention to offer VPT, and are there differences in terms of number of statements and relevance ratings between perceived advantages and disadvantages of VPT?

#### 1.2.2. Desired learning opportunities

Not only the perceived advantages and disadvantages of VPT, but also desired learning opportunities for VPT, could influence students’ future behavior (*cf.* Cai et al., 2008; Adukaite et al., 2017; McBride et al., 2020). Research in the field of teaching examined the relationship between learning opportunities and behavioral performance, indicating that the more learning opportunities there are, the more likely the corresponding behavior will be performed (*cf.* Parise and Spillane, 2010). Due to the novelty of VPT, little to no learning opportunities for VPT are currently implemented in study programs in Germany. Therefore, it is important to ask psychology students about their desired learning opportunities regarding this topic. Similarly, Buckman et al. (2021) asked in-service mental healthcare professionals about their training needs or required support to deliver VPT more effectively. Among other things, they reported the need for learning opportunities about research evidence for the effectiveness of VPT, training on the functions of the platforms used for video sessions and differences between platforms, as well as technical support and role-playing. But there is no research with psychology students on this topic yet. Therefore, the following research question was formulated:

*RQ2*: Which learning opportunities do psychology students desire in their study program to feel able to offer VPT after graduation, and how relevant do they rate these learning opportunities in relation to their intention to offer VPT?

It is conceivable that differences in VPT knowledge between students in bachelor’s and master’s programs may impact how they perceive learning opportunities for VPT (*cf.* König et al., 2018). In the field of teacher education, disparities in learning opportunities and knowledge have been observed between bachelor’s and master’s students. For example, Tachtsoglou and König (2017) found that master’s students possessed a greater depth of pedagogical knowledge compared to their bachelor’s counterparts. Similar variations could exist among psychology students. Furthermore, the temporal proximity of participants’ experience in using VPT as licensed psychotherapists may influence their preferences and desires. This variation in perception may be manifested in the content and relevance ratings of the statements, as psychology students in bachelor’s and master’s programs may hold divergent perspectives (either more or less detailed) regarding their future role as licensed psychotherapists. These considerations led to the following research question:

*RQ3*: Are there differences between psychology students in bachelor’s versus master’s study programs regarding desired learning opportunities in terms of types of learning opportunities, number of named learning opportunities, and relevance ratings?

## 2. Materials and methods

### 2.1. Participants

The final data set included 255 German-speaking psychology students of legal age (218 women, 85.5%) with a mean age of 25.74 years (*SD* = 5.60, range = 18–52). One participant was previously excluded because they asked not to consider the given answers. Non-psychology students and psychology students who planned not to become psychotherapists after graduation were not permitted to participate and were immediately directed to the final page of the survey. The final data set consisted of 140 (54.9%) psychology students in bachelor’s program and 115 (45.1%) in master’s program of psychology. The most frequently selected psychotherapy approach that the psychology students intended to offer as licensed psychotherapist was behavioral psychotherapy (*n* = 152, 59.6%), followed by depth-psychology oriented psychotherapy (*n* = 45, 17.6%), systemic psychotherapy (*n* = 34, 13.3%), analytical psychotherapy (*n* = 15, 5.9%), and others (*n* = 9, 3.5%).

Participants were recruited through convenience and snowball sampling. The link to the online study was disseminated to psychology students via mailing lists of German universities, social media, the survey platform of a national journal (Psychologie Heute), and via lecturers in psychology courses. Participation in the study was voluntary and no incentives were provided. No identifying data were collected to guarantee the anonymity of participants. At the beginning of the study, participants were informed about the study’s aim, the anonymity of their data, that all data would be processed only for research purposes, and that they could stop the study at any time. The participants provided informed consent. The online study ran for 103 days, beginning July 13, 2021.

### 2.2. Procedure

We collected the data using an online survey software “Unipark” (Tivian XI GmbH). First, participants indicated whether they were studying psychology and whether they intended to become a psychotherapist after graduation. Subsequently, they provided their age, gender, which psychotherapy approach they plan to offer as licensed psychotherapist, study stage, and how many semesters they have studied psychology to date. Then, they read our definition of VPT and what they should consider when answering the questions to ensure the validity of responses. We highlighted the following five aspects:

1. VPT was defined as psychotherapy conducted online using a video platform with a video display and an audio connection. The psychotherapist and the patient would not be sitting in the same room. The patient would usually be at home. However, both could see and hear each other via the medium used.
2. No distinction between the different forms of psychotherapy (e.g., analytical psychotherapy, behavioral therapy) should be made when answering the questions. It should be basically about VPT.
3. VPT should be understood as an additional offer that psychotherapists could provide if a normal face-to-face treatment (one, several, or all sessions) was not possible for various reasons or if VPT was preferred.
4. The participants were asked not to think about specific disorder characteristics of patients. The focus was on VPT (in addition to face-to-face psychotherapy) – independent of disorder patterns.
5. Participants should assume that all legal and technical conditions necessary for conducting VPT are in place (e.g., acceptance by health insurance companies, data security, and suitable software).

Afterwards, participants were asked several questions about their attitudes toward VPT, perceived subjective norm, expected usefulness of VPT, satisfaction with video conferencing, general self-efficacy, self-efficacy in video conferencing, and the intention to offer VPT when they become licensed psychotherapists. In the qualitative part of the study, open-ended questions were asked about perceived advantages and disadvantages of VPT, as well as desired learning opportunities regarding VPT in the study program. The median study duration was 10.92 minutes.

### 2.3. Quantitative measures

Due to lack of availability of validated scales in this under-researched topic, we had to adapt some scales to the context of our study. Additionally, we translated some established scales into German (translate-translate back method). The following sections describe the measures used in detail.

#### 2.3.1. Attitudes toward VPT

Attitudes toward VPT were measured with a scale of Doran and Lawson (2021) about attitudes toward telemental health, which we translated for the present study. The scale comprises seven items (e.g., “I have positive feelings about video psychotherapy,” Cronbach’s α = 0.88). The five answer options are “strongly disagree,” “somewhat disagree,” “neither nor,” “somewhat agree,” and “strongly agree,” without numerical markers.

#### 2.3.2. Subjective norms

Based on a scale used by Ajzen (2006) for his Theory of Planned Behavior, we adapted three items to assess the perceived (subjective) norm regarding the offer of VPT (e.g., “People I care about think I should offer VPT in the future,” α = 0.96). The scale ranges from 1 (“strongly disagree”) to 7 (“strongly agree”), with numerical but without verbal markers between the endpoints of the scale.

#### 2.3.3. Expected usefulness of VPT

Expected usefulness of VPT was assessed with a translated and adapted scale of Gidenko (2017) which originally measured perceived usefulness of telemental health services. We used four of the five items, because one item (“Telemental health care services would make it easier to make referrals for mental health care”) did not fit in the context of our study. The final scale comprises four items (e.g., “Video psychotherapy could make patient care easier,” α = 0.84). The five answer options are “strongly disagree,” “disagree,” “neutral,” “agree,” and “strongly agree,” without numerical markers.

#### 2.3.4. Satisfaction with video conferencing

We measured satisfaction with video conferencing with five items translated and adapted from Chao (2019), originally referring to satisfaction with mobile learning. Participants indicated their satisfaction regarding their experiences with video conferencing in the last year (e.g., “I was very pleased with video conferencing,” α = 0.90). The scale ranges from 1 (“strongly disagree”) to 5 (“strongly agree”), with numerical but without verbal markers between the endpoints of the scale.

#### 2.3.5. General self-efficacy

General self-efficacy was measured using the original short scale by Beierlein et al. (2012). The German short scale consists of three items (e.g., “In difficult situations I can rely on my abilities,” α = 0.84). The scale uses a five-point format from 1 (“not true at all”), 2 (“barely true”), 3 (“somewhat true”), 4 (“quite true”), to 5 (“completely true”). The original 10-item scale has a reliability of α = 0.92 (Schwarzer and Jerusalem, 1999), thus using the short scale is associated with an acceptable loss of reliability.

#### 2.3.6. Self-efficacy in video conferencing

To measure self-efficacy in video conferencing, we translated a corresponding subscale by Giroux and Lachance (2008) from French into German (e.g., “I feel competent in the use of video conferencing,” α = 0.86). The scale comprises five items and participants indicated the extent to which the statements applied to them on a scale from 1 (“not true at all”) over 4 (“somewhat true”) to 7 (“completely true”), with continuous numerical markers.

#### 2.3.7. Intention to offer VPT

To measure the intention to offer VPT as licensed psychotherapist, we translated and slightly adapted three items from a scale by Venkatesh and Bala (2008) on behavioral intention (e.g., “Assuming I had the opportunity to offer video psychotherapy, I intend to offer it,” α = 0.95). The scale uses a seven-point format from 1 (“strongly disagree”), 2 (“disagree”), 3 (“somewhat disagree”), 4 (“neutral (neither nor)”), 5 (“somewhat agree”), 6 (“agree”), to 7 (“strongly agree”).

### 2.4. Qualitative measures and coding procedure

A qualitative approach was used to investigate advantages and disadvantages of VPT, as well as desired learning opportunities for VPT. Therefore, the quantitative measures were followed by open-ended questions to assess participants’ perceived advantages and disadvantages of VPT (“Please list up to five aspects that you perceive as advantages of video psychotherapy” and “Please list up to five aspects that you perceive as disadvantages of video psychotherapy”), as well as desired learning opportunities (“Please list up to five learning opportunities (learning content and/or experiences) that you would want to have in your psychology studies to feel well prepared for video psychotherapy as a licensed psychotherapist”). The participants could write down up to five statements per question. Responses were limited to 150 characters per statement in order to support the formulation of the quintessence of each response. Following the method outlined by Hoss et al. (2021), participants additionally indicated how relevant the named advantage or disadvantage is in relation to their personal intention to offer VPT as licensed psychotherapists, and how relevant a named learning opportunity is to feeling well-prepared to perform VPT (1 = “hardly relevant” to 5 = “very relevant”). Only if a relevance rating referred to a valid statement, we included it in further analyses. The order of the qualitative questions regarding perceived advantages and disadvantages of VPT was randomized across participants to counteract order effects. The question on desired learning opportunities was always asked last to enable the participants to transfer their mentioned advantages and disadvantages directly into concrete desired learning opportunities.

We analyzed the qualitative data using content analysis based on the standard approach developed by Mayring (2015) and following a previous work of Hoss et al. (2021): First, we read all qualitative statements and condensed them to extract the key messages, if necessary. If multiple statements were provided in one answer field, only the first statement was considered, and the others were disregarded for further analyses. Second, we inductively created a category system based on about 10% of the given data for each of the three topics. Subsequently, we calculated the inter-coder reliability between the two raters after coding approximately 10% of the material to identify any potential sources of error and to optimize the category system accordingly. In this sense, an iterative process based on all qualitative data led to the final category system. Using the final category system, two independent coders coded all the data, and inter-coder reliability was calculated using Kappa (Cohen, 1960). In cases of disagreement between coders, a mutually agreeable solution was subsequently found via discussion between the two coders. The category system for perceived advantages of VPT contained 17 categories (κ = 0.96), perceived disadvantages of VPT were assigned to 18 categories (κ = 0.95), and desired learning opportunities for VPT in psychology studies were assigned to 14 categories (κ = 0.97). There was an additional residual category for each topic for statements that did not fit into any of the other categories.

## 3. Results

We first present the results of the hypotheses-driven quantitative part of the study (Section 3.1) and afterwards the explorative qualitative results (Section 3.2). We ran all analyses with SPSS version 28.0.

### 3.1. Intention to offer VPT (H1–H7)

We conducted a multiple regression analysis to test the seven hypotheses regarding psychology students’ intention to offer VPT. All statistical assumptions were checked before calculating the analyses (cf. Poole and O’Farrell, 1971). We found no outliers, multicollinearity, or autocorrelation, but linearity and normality in the regression model. Because of small hints for heteroscedasticity, we applied bootstrapping method (5,000 iterations; bias-corrected and accelerated) for significance testing (cf. Hayes and Cai, 2007). The multiple regression model comprised intention to offer VPT as the dependent variable and attitudes toward VPT (H1), subjective norm (H2), expected usefulness of VPT (H3), satisfaction with current video conferencing (H4), general self-efficacy (H5), self-efficacy in video conferencing (H6) and study stage (bachelor’s or master’s program; H7) as independent variables. Although we formulated the hypotheses mostly one-sided, two-sided *p*-values are reported (*cf.* Kimmel, 1957; Koch, 1991).

First, we calculated correlations between all independent variables. As shown in Table 1, these correlations were low to medium with few exceptions. The two highest positive correlations were between expected usefulness of VPT and attitudes toward VPT (*r* = 0.66, *p* < 0.001), and between satisfaction with video conferencing and attitudes toward VPT (*r* = 0.61, *p* < 0.001). We found no significant negative correlations between the independent variables. Altogether, the strength and direction of the correlations were as expected, indicating construct validity.

**Table 1:** Descriptive statistics and bivariate correlations (Pearson *r*) among all independent variables of the multiple regression model.

		*M*	*SD*	Correlations					
				1.	2.	3.	4.	5.	6.
1.	Attitudes toward VPT^1^	3.60	0.75						
2.	Subjective norm^2^	3.70	1.53	0.49***					
3.	Expected usefulness of VPT^1^	4.21	0.64	0.66***	0.35***				
4.	Satisfaction with video conferencing^1^	3.37	0.87	0.61***	0.34***	0.49***			
5.	General self-efficacy^1^	4.11	0.57	0.20**	0.15*	0.22***	0.23***		
6.	Self-efficacy in video conferencing^2^	5.70	0.99	0.22***	0.17**	0.33***	0.35***	0.38***	
7.	Study stage (bachelor vs. master)^3^	–	–	−0.03	0.04	−0.02	0.03	0.16**	0.21***

^1^The scale ranged from 1 to 5. ^2^The scale ranged from 1 to 7. ^3^Study stage was dummy coded (0 = bachelor, 1 = master). ***two-tailed *p* < 0.001, **p < 0.01, *p < 0.05.

Table 2 shows the results of the multiple regression analysis and the bivariate correlations between independent variables and the dependent variable of the research model. All independent variables, except for study stage, showed a significant positive correlation with the intention to offer VPT. The strongest positive correlation was between attitudes toward VPT and intention to offer VPT (*r* = 0.80, *p* < 0.001). The multiple regression model explained 73.0% of inter-individual variance, *F*(7, 247) = 95.64, *p* < 0.001. Four of the seven independent variables were significant in the regression model: Attitudes toward VPT (β = 0.45, *p* < 0.001) was positively related to intention to offer VPT and it was the most relevant independent variable as indicated by the standardized regression coefficient. Moreover, subjective norm (β = 0.16, *p* < 0.001) showed a significant positive relationship with the intention to offer VPT, as well as expected usefulness of VPT (β = 0.25, *p* < 0.001), and satisfaction with video conferencing (β = 0.17, *p* < 0.001). In contrast, self-efficacy in video conferencing (β = 0.05, *p* = 0.294), general self-efficacy (β = −0.06, *p* = 0.128) and study stage (β = −0.03, *p* = 0.348) were not significantly related to intention of offer VPT. Although both self-efficacy variables did not become significant in the multiple regression, they showed a significant bivariate correlation with intention to offer VPT (Table 2). In sum, the four variables taken from the two established models (Theory of Planned Behavior and Technology Acceptance Model) explained a considerable amount of variance in the intention to offer VPT.

**Table 2:** Bivariate correlations between independent and dependent variables of the research model and the results of the multiple regression analysis.

Independent variable	*r*^a^	*p^b^*	*B*^c^	β	*p*^d^
Attitudes toward VPT	0.80	<0.001	0.90 [0.68–1.11]	0.45	<0.001
Subjective norm	0.53	<0.001	0.16 [0.08–0.24]	0.16	<0.001
Expected usefulness of VPT	0.69	<0.001	0.57 [0.28–0.84]	0.25	<0.001
Satisfaction with video conferencing	0.62	<0.001	0.29 [0.14–0.44]	0.17	<0.001
General self-efficacy	0.16	0.009	−0.15 [−0.35–0.04]	−0.06	0.128
Self-efficacy in video conferencing	0.29	<0.001	0.08 [−0.07–0.23]	0.05	0.294
Study stage (bachelor vs. master)	−0.04	0.555	−0.09 [−0.28–0.11]	−0.03	0.348

Study stage was dummy coded (0 = bachelor, 1 = master). ^a^ Bivariate correlations between independent variable and the dependent variable of the multiple regression model (Pearson *r*). ^b^ *p*-values of bivariate correlations. ^c^ *B*-values representing unstandardized coefficients of the multiple regression model and their 95% confidence intervals (bootstrapping with 5,000 iterations). ^d^ *p*-values of independent variables within multiple regression are based on bootstrapping with 5,000 iterations.

### 3.2. Qualitative content analyses

In total, the participants provided 2,314 statements regarding advantages and disadvantages of VPT and desired learning opportunities. We categorized these statements via content analysis (*cf.* Mayring, 2015). Detailed definitions and explanations of the category system can be found in the Supplementary material, elucidating the specific criteria that guided the classification process. These definitions serve as a reference point for understanding the inclusion criteria for statements within their respective categories. The following section provides an explanation of the results regarding the advantages and disadvantages (Section 3.2.1) and the desired learning opportunities (Section 3.2.2).

#### 3.2.1. Perceived advantages and disadvantages of VPT (RQ1)

In total, participants provided 839 statements describing advantages of VPT. Table 3 presents the classification system covering 17 categories (plus one residual category). All statements were assigned to one of these categories to allow quantitative analyses. The most frequently mentioned advantages of VPT concerned easier access to or easier delivery of psychotherapy using VPT, such as *low inhibition threshold* (114 statements, 13.6% of all statements), *reduced mental and physical barriers* (72, 8.6%), *protection against disease and pandemic* (70, 8.3%), *accessibility* (68, 8.1%), *availability of the psychotherapist* (33, 3.9%), *lower costs* (24, 2.9%), general *simplicity* of VPT (20, 2.4%), *anonymity* (11, 1.3%), and *documentation capabilities* (7, 0.8%). Many of the mentioned advantages of VPT also described local flexibility between the psychotherapist and the patient: *flexibility in terms of location* (94 statements, 11.2%), and *no travel necessary* (85, 10.1%). Moreover, participants mentioned broader psychotherapeutic care via VPT like *psychotherapeutic care in rural areas* (20, 2.4%) and *psychotherapeutic care worldwide* (19, 2.3%). Further, statements about *general flexibility* (82, 9.8%), as well as *time-related benefits* (63, 7.5%), *distance-related benefits* (7, 0.8%), and the *spirit of the age* (9, 1.1%) of VPT as a method of delivering psychotherapy were mentioned. 41 of all statements (4.9%) did not fit in any category and were assigned to the residual category. In some cases, several statements of one participant were assigned to the same category. Importantly, adjusting the data for such multiple mentions did not result in any major changes in the order of the categories according to the frequency of statements, as shown in Table 3.

**Table 3:** Mentioned advantages of VPT, divided into the defined categories with relevance rating and example statements.

Category of advantages	Number of statements	*M*^a^	*SD*^a^	Concise description of category^b^	Example statements of category
1. Low inhibition threshold	114 (103)	4.07***	0.90	Low inhibition threshold, easier start and comfort in a familiar environment.	“inhibition threshold for psychotherapy lower”
2. Flexibility in terms of location	94 (87)	4.31***	0.75	Flexible location, no need to change therapists or interrupt therapy during relocation or vacations.	“location independence,” “same therapist after the change of location”
3. No travel necessary	85 (78)	3.79***	0.98	Elimination of travel distances, lower costs, time savings, and environmental benefits.	“time savings due to the elimination of travel time,” “no long journeys for patients”
4. General flexibility	82 (75)	4.06***	0.89	General and individual flexibility, including coordination with patients.	“increased individual adaptation, to the needs of the patient,” “home office”
5. Reduced mental and physical barriers	72 (64)	4.58***	0.60	Reduced barriers, suitable for various disorders and patients.	“barrier-free for the physically impaired,” “people who care for others can participate in therapy,”
6. Protection against disease and pandemic	70 (64)	4.30***	0.86	Psychotherapeutic care during illness or pandemics, including protection and home-based treatment.	“under COVID-19 pandemic, good care,” “in cases of illness, a substitute”
7. Accessibility	68 (64)	4.46***	0.72	Improved accessibility, increased capacity, shorter waiting times, and better patient care.	“shorter waiting times,” “easy accessibility”
8. Time-related benefits	63 (57)	3.95***	0.94	Time-related benefits, flexible scheduling, and time savings.	“better integration into the patient’s everyday life”
9. Availability of the psychotherapist	33 (32)	4.21***	0.86	Availability of psychotherapists, better access and emergency support.	“fast availability,” “better emergency care”
10. Lower costs	24 (24)	3.54	1.29	Cost savings, reduced expenses and overhead.	“fewer rent costs for therapists,” “cost-saving”
11. Simplicity	20 (20)	3.75**	1.07	Simplicity of VPT, ease of use and reduced effort.	“easier handling for patients,” “uncomplicated”
12. Psychotherapeutic care in rural areas	20 (19)	4.60***	0.82	Access to mental health care in rural and isolated regions.	“providing care in rural areas,” “providing care to patients in remote regions”
13. Psychotherapeutic care worldwide	19 (18)	4.32***	0.82	International availability of psychotherapy, including during stays abroad.	“accessibility for patients abroad,” “therapy in the native language possible from abroad”
14. Anonymity	11 (11)	3.73	1.19	Positive aspects of anonymity, reduced stigmatization, and enhanced privacy.	“less stigmatization of patient by other people”
15. Spirit of the age	9 (9)	3.56	1.24	Modern and state-of-the-art, meeting patient preferences.	“modernization of the health care system”
16. Documentation capabilities	7 (7)	3.43	0.98	Documentation capabilities, recording and reviewing sessions.	“eventual video recording facilitates documentation,” “documentability by recording the session”
17. Distance-related benefits	7 (6)	3.86*	0.69	Interpersonal distance, maintaining a professional distance.	“professional distance can be better maintained”
18. Residual category	41 (33)	3.85***	1.04	Miscellaneous statements that do not fit into the defined categories.	“less good patient contact,” “better than nothing”

Column “number of statements” shows the total number of statements and in brackets the number of participants who provided at least one statement of the respective category (i. e., corrected for multiple responses of individual participants falling into the same category). ^a^ For relevance ratings, one-sample *t*-tests were computed against the scale’s midpoint of 3, ***p < 0.001, **p < 0.01, *p < 0.05. ^b^ For a more detailed description of the categories, please refer to the Supplementary material.

The mean relevance ratings of each category were above the scale’s midpoint of 3. The grand mean (across all relevance ratings) for perceived advantages of VPT was significantly above the scale’s midpoint (*M* = 4.13, *SD* = 0.92), *t*(838) = 35.48, *p* < 0.001, *d* = 1.23. The five categories of advantages ranked most relevant on average were *psychotherapeutic care in rural areas*, *reduced mental and physical barriers*, *accessibility*, *psychotherapeutic care worldwide*, and *flexibility in terms of location*. All statistical results are presented in Table 3.

In addition to the statements about advantages of VPT, psychology students mentioned 898 statements concerning disadvantages of VPT. Table 4 presents the classification system covering 18 categories (plus one residual category). The most frequently mentioned disadvantages of VPT concerned problems during a psychotherapy session: *lack of closeness between patient and therapist* (113, 12.6% of all 898 statements), *lack of nonverbal cues via video* (112, 12.5%), *problems with technology or internet connection* (98, 10.9%), that *therapeutic relationship suffers* (70, 7.8%), and that there is *no safe space for patients* (51, 5.7%). The possibility of *communication problems and misunderstandings* (40, 4.5%), that *more distraction is possible* (38, 4.2%), and that *showing and recognizing empathy and emotions is difficult* (34, 3.8%) also addressed potential problems during the psychotherapy session. Moreover, statements about limited application possibilities were given, like *technology as a prerequisite* (66, 7.3%), that VPT is *not appropriate for all disorders and patients* (46, 5.1%) and *not appropriate for all therapeutic methods* (45, 5.0%), that VPT can be of *lower efficacy and less effectiveness* (25, 2.8%), and allows *less opportunities for intervention* (14, 1.6%). Furthermore, participants stated *lower commitment and motivation of patients* (39, 4.3%), *no benefits from leaving home* (34, 3.8%), *risk for data privacy* (22, 2.4%), *higher cognitive effort* (10, 1.1%), and a *more difficult organization and bureaucracy* (9, 1.0%) as disadvantages of VPT. 32 statements (3.6%) did not fit in any category and were assigned to the residual category. Again, adjusting the data for multiple responses of a person did not result in any major changes in the results.

**Table 4:** Mentioned disadvantages of VPT, divided into the defined categories with relevance rating and example statements.

	Category of disadvantages	Number of statements	*M*^a^	*SD*^a^	Concise description of category^b^	Example statements of category
1	Lack of closeness between patient and psychotherapist	113 (103)	4.37***	0.80	Spatial distance and lack of personal closeness between patient and psychotherapist.	“distance from the patient,” “more impersonal,” “no shared experience”
2	Lack of nonverbal cues	112 (103)	4.41***	0.72	Limited nonverbal communication cues.	“less perception of non-verbal cues,” “only face visible”
3	Problems with technology or internet connection	98 (94)	4.03***	0.91	Connection problems and disruptions during VPT sessions.	“patient’s technology problems interfere with VPT,” “Internet connection problems”
4	Therapeutic relationship suffers	70 (64)	4.60***	0.65	Challenges in establishing and maintaining the therapeutic relationship.	“worse relationship with each other,” “relationship building with the patient more difficult”
5	Technology as a prerequisite	66 (55)	3.82***	1.11	Dependency on technical devices and internet access.	“technical knowledge required,” “technology is costly”
6	No safe space for patients	51 (47)	4.27***	0.80	Lack of a safe and private environment for patients.	“protected space not guaranteed at patient’s home”
7	Not appropriate for all disorders and patients	46 (40)	3.87***	1.00	Unsuitability or limitations for certain disorders or patients.	“technology acquisition as a prerequisite excludes people”
8	Not appropriate for all therapeutic methods	45 (42)	4.36***	0.71	Incompatibility with certain therapeutic methods.	“limitation of treatment methods”
9	Communication problems and misunderstandings possible	40 (38)	4.33***	0.83	Disrupted communication and interaction during VPT sessions.	“higher probability of misunderstanding,” “loss of information”
10	Lower commitment and motivation of patients	39 (36)	3.72***	0.92	Perception of VPT as less obligatory for patients.	“invites to a quicker termination of the therapy session,” “higher non-commitment”
11	More distraction possible	38 (37)	3.97***	0.92	Increased distractions and interference.	“distraction at home,” “being undisturbed at home can be difficult”
12	No benefits from leaving home	34 (32)	4.09***	0.83	Absence of the benefits associated with leaving the house for face-to-face psychotherapy.	“no need to leave the house,” “getting out of the daily routine is missing”
13	Showing and recognizing empathy and emotions is difficult	34 (32)	4.59***	0.61	Difficulty in expressing and perceiving empathy and emotions.	“empathy is difficult to convey,” “reduced empathy”
14	Lower efficacy and less effectiveness	25 (22)	4.24***	0.72	Potentially lower effectiveness or efficiency compared to face-to-face psychotherapy.	“intimate conversations better in presence,” “only limited diagnosis possible”
15	Risk for data privacy	22 (22)	3.82***	0.85	Concerns regarding data security during VPT.	“feeling of lack of data protection,” “data protection concerns”
16	Less opportunities for intervention	14 (14)	4.57***	0.65	Limited opportunities for intervention and interaction for psychotherapists.	“less possibilities of intervention,” “in case of emergency the person is not present”
17	Higher cognitive effort	10 (10)	4.00**	0.82	Increased effort and cognitive load during VPT.	“cognitive strain of platform use,” “straining to concentrate”
18	More difficult organization and bureaucracy	9 (9)	3.89	1.17	Additional organizational and bureaucratic complexities for psychotherapists offering VPT.	“health insurance approval not guaranteed,” “organization of appointments more difficult”
19	Residual category	32 (27)	4.09***	0.96	Miscellaneous statements that do not fit into the defined categories.	“people are on screens too much anyway”

Column “number of statements” shows the total number of statements and in brackets the number of participants who provided at least one statement of the respective category (i. e., corrected for multiple responses of individual participants falling into the same category). ^a^ For relevance ratings, one-sample *t*-tests were computed against the scale’s midpoint of 3, ***p < 0.001, **p < 0.01. ^b^ For a more detailed description of the categories, please refer to the Supplementary material.

Regarding the relevance rating of disadvantages, all categories were rated above the scale’s midpoint and, except for one, these deviations from the midpoint were statistically significant, see Table 4. Similar to the perceived advantages, the grand mean of all relevance ratings for perceived disadvantages of VPT was above the scale’s midpoint of 3 (*M* = 4.20, *SD* = 0.87), *t*(897) = 41.22, *p* < 0.001, *d* = 1.38. The five categories ranked most relevant on average were: *therapeutic relationship suffers, showing and recognizing empathy and emotions is difficult*, *less opportunities for intervention*, *lack of nonverbal cues*, and *lack of closeness between patient and psychotherapist*.

To find out if there were differences between the perceived advantages and disadvantages, we compared the number of statements as well as the relevance ratings using *t*-tests for paired samples. We found a significant difference between the mean number of mentioned advantages per participant (*M* = 3.29, *SD* = 1.50) and the mean number of disadvantages (*M* = 3.52, *SD* = 1.55), *t*(254) = −2.70, *p* = 0.008, *d* = −0.17. Disadvantages were stated more often, but the effect size was small. There was no significant difference between perceived advantages (*M* = 4.14, *SD* = 0.60) and disadvantages of VPT (*M* = 4.20, *SD* = 0.60) regarding mean relevance ratings per participant, *t*(228) = −1.19, *p* = 0.237, *d* = −0.08. Hence, although slightly more disadvantages were mentioned per person, disadvantages and advantages of VPT were perceived as equally relevant.

#### 3.2.2. Desired learning opportunities for VPT in psychology studies (RQ2)

Overall, 577 statements were written about desired learning opportunities for VPT in the study program of psychology students. Table 5 presents the classification system covering 14 categories (plus one residual category). The categories address two main areas of desired learning opportunities. On the one hand, participants desired training and practical experiences, on the other hand, they desired information about VPT. The desired learning opportunities for VPT related to training included *training for technical skills* (100, 17.3% of all 577 statements), *practical application (role-playing/self-experience)* (70, 12.1%), *training for conversational techniques via video conferencing* (47, 8.1%), *training to build therapeutic relationship via video conferencing* (36, 6.2%), *insights into the practice of VPT* (30, 5.2%), *training to recognize and showing nonverbal cues via video conferencing* (21, 3.6%), and *training to increase motivation and compliance of patients* (9, 1.6%). Desired information about VPT was divided into *general information about VPT* (60, 10.4%), *information on (health insurance) legal requirements* (47, 8.1%), *information on appropriate methods for VPT* (32, 5.5%), *information on handling difficult situations during VPT sessions* (27, 4.7%), *information on efficacy studies on VPT* (23, 4.0%), *information on opportunities and limitations of VPT* (23, 4.0%), and *information on appropriate disorders and patients* (14, 2.4%). Again, adjusting the data for multiple responses did not result in any major changes in the results, as shown in Table 5.

**Table 5:** Mentioned desired learning opportunities, divided into the defined categories with relevance rating and example statements.

	Category of desired learning opportunities	Number of statements	*M*^a^	*SD*^a^	Concise description of category^b^	Example statements of category
1	Training for technical skills	100 (93)	4.04***	0.92	Training for technical skills and software/hardware proficiency for VPT as well as practicing the use or acquiring technology-related skills.	“handling programs for video psychotherapy should be taught”
2	Practical application (role-playing/self-experience)	70 (60)	4.57***	0.63	Desires for role-playing and self-experience to simulate and learn about VPT in a practical way.	“role plays in online format,” “self-experiences using video psychotherapy”
3	General information about VPT	60 (50)	4.23***	0.83	General information about VPT, including delivery methods and differences from face-to-face therapy.	“general information about video psychotherapy,” “instructions on how to organize the online setting”
4	Training for conversational techniques via video conferencing	47 (46)	4.23***	0.84	Training on conducting effective conversations and maintaining communication flow in VPT.	“learning conversation techniques,” “practicing dialogue guidance in clinical psychology via video”
5	Information on (health insurance) legal requirements	47 (45)	4.40***	0.71	Information on legal issues and health insurance considerations for VPT such as privacy or data protection, legal specifics, and health insurance legal accounting	“information on data protection,” “legal circumstances”
6	Training to build therapeutic relationship via video conferencing	36 (34)	4.69***	0.47	Training to build a therapeutic relationship via videoconferencing including aspects of showing empathy and encouragement or recognizing feelings.	“therapeutic relationship in the digital setting,” “empathy through video”
7	Information on appropriate methods for VPT	32 (26)	4.31***	0.86	Information on specific methods of VPT and if they may or may not be feasible.	“methods specific to video psychotherapy,” “learning method diversity online”
8	Insights into the practice of VPT	30 (24)	4.33***	0.76	To learn with experiential reports from and conversations with video psychotherapists and patients, as well as watching sample videos of VPT.	“invite psychotherapists in seminar,” “interviews from therapists with experience in online therapy”
9	Information on handling difficult situations during VPT sessions	27 (25)	4.33***	0.92	Information on dealing with difficult situations in VPT, such as dissociation or technical problems.	“dealing with disconnection,” “learning appropriate behaviors when patients dissociate”
10	Information on efficacy studies on VPT	23 (23)	4.43***	0.84	Information on effectiveness of VPT and related studies including studies on methodological aspects.	“studies on effectiveness of video therapy,” “studies on virtual therapy groups”
11	Information on opportunities and limitations of VPT	23 (21)	4.52***	0.67	Information on advantages, disadvantages, and limitations of VPT, including those related to videoconferencing.	“discussions about advantages and disadvantages,” “possibilities and limitations of video therapy”
12	Training to recognize and showing nonverbal cues via video conferencing	21 (19)	4.33***	0.73	Training to convey nonverbal cues, such as facial expressions, body language, and gestures, in a virtual setting, as well as the desire to develop the ability to accurately interpret and recognize these cues.	“learning to interpret facial expressions,” “nonverbal communication in the online setting”
13	Information on appropriate disorders and patients	14 (13)	4.50***	0.94	Information on appropriate disorders and patients related to the question for which disorders/patients VPT is more appropriate and for which or whom it is less appropriate.	“video therapy for various disorders,” “information about usability for specific disorders”
14	Training to increase motivation and compliance of patients	9 (9)	4.00*	1.00	Training to enhancing client motivation and compliance in VPT.	“learning patient motivation,” “compliance”
15	Residual category	38 (30)	4.32***	0.87	Miscellaneous statements that do not fit into the defined categories.	“distinction between therapy and counseling”

Column “number of statements” shows the total number of statements and in brackets the number of participants who provided at least one statement of the respective category (i. e., corrected for multiple responses of individual participants falling into the same category). ^a^ For relevance ratings, one-sample *t*-tests were computed against the scale’s midpoint of 3, ***p < 0.001, *p < 0.05. ^b^ For a more detailed description of the categories, please refer to the Supplementary material.

Regarding the relevance rating of desired learning opportunities, all categories received significantly higher ratings than the scale’s midpoint. The grand mean of relevance ratings (*M* = 4.33, *SD* = 0.81) was above the scale midpoint of 3, *t*(576) = 39.24, *p* < 0.001, *d* = 1.63. The five highest ratings regarding desired learning opportunities were the following: *training to build therapeutic relationship via video conferencing*, *practical application (role-playing/self-experience)*, *information on opportunities and limitations of VPT*, *information on appropriate disorders and patients*, and *information on efficacy studies on VPT*, see Table 5.

#### 3.2.3. Comparison of learning opportunities desired by psychology students in a bachelor’s versus master’s program (RQ3)

First, to investigate whether psychology students in bachelor’s and master’s program provided different numbers of statements regarding learning opportunities, we calculated a *t*-test for independent samples with (mean) number of statements per person as dependent variable and study stage as factor. Psychology students in the bachelor’s program reported on average 2.21 (*SD* = 1.68) learning opportunities and psychology students in master’s program reported 2.32 (*SD* = 1.71). This small difference was not statistically significant, *t*(253) = −0.50, *p* = 0.614, *d* = −0.06.

Second, to identify possible differences in content, we compared the top five most frequently mentioned statements between psychology students in the bachelor’s and master’s programs. In general, there were no significant discrepancies observed between the statements of these two groups, as shown in Table 6. However, there were some slight differences that are worth mentioning. Specifically, among all statements made by psychology students in a bachelor’s program, 10.0% related to the desire for *training for conversational techniques via video conferencing*, whereas this category was not in top five of students in a master’s program (6.0%). Conversely, 7.9% of the statements of master students were related to the desire for *information on appropriate methods for VPT*, which did not appear in the top five of bachelor students (3.5%).

**Table 6:** Comparison of bachelor and master students’ five most mentioned categories for desired learning opportunities for VPT in psychology studies.

	Most mentioned desired learning opportunities for VPT	
	Bachelor	Master
1.	Training for technical skills (18.7%)	Training for technical skills (15.7%)
2.	Practical application (role-playing/self-experience) (12.6%)	Practical application (role-playing/self-experience) (11.6%)
3.	General information about VPT (10.6%)	General information about VPT (10.1%)
4.	Training for conversational techniques via video conferencing (10.0%)	Information on (health insurance) legal requirements (8.2%)
5.	Information on (health insurance) legal requirements (8.1%)	Information on appropriate methods for VPT (7.9%)

The numbers in brackets indicate the percentage of all statements that were categorized in the corresponding category.

Third, we computed a *t*-test for independent samples with the relevance ratings of desired learning opportunities as dependent variable and study stage as factor. We found no significant difference, *t*(200) = −1.15, *p* = 0.254, *d* = −0.16.

To sum up, our analyses did not reveal major differences in the desired learning opportunities between psychology students enrolled in bachelor’s and master’s programs.

## 4. Discussion

This mixed-methods study had two main objectives: First, we investigated which person-related variables of psychology students are related to their intention to offer VPT after graduation (Section 4.1). Second, we intended to explore psychology students’ perceptions of advantages and disadvantages of VPT (Section 4.2) and to identify desired learning opportunities within the study program (Section 4.3). In the following, the results of the study are summarized and integrated into existing literature. Additionally, we discuss theoretical and practical implications and limitations of the present study.

### 4.1. Intention to offer VPT

Using a quantitative approach, our research model explains a total of 73% of the inter-individual variance in the intention of psychology students to offer VPT. This amount of variance explanation is very high for psychological studies (*cf.* Cohen, 1988). In this context, the attitude toward VPT showed the strongest relationship with intention, followed by expected usefulness, subjective norm, and satisfaction with video conferencing. In contrast, both self-efficacy variables and study stage showed no significant relation to intention in the multiple regression analysis.

Attitudes toward VPT showed divergent results in the literature. For example, Monthuy-Blanc et al. (2013) found that in-service psychotherapists’ attitudes toward telemental health were not a significant predictor of intention. However, Gidenko (2017) found that attitudes toward telemental health are the most important determinant of the intention of primary care physicians to use technology for care services. This is consistent with the finding that the intention of healthcare professionals to use e-health technology applications is significantly related to their attitudes, among other variables (Zayyad and Toycan, 2018). In line with these findings, we observed that attitude showed the largest positive relation with intention to offer VPT (H1) in our study. Expected usefulness of VPT showed the second-largest relation with intention (H3), which aligns with the findings of Monthuy-Blanc et al. (2013), whose model showed that perceived usefulness had the strongest relation to the intention of psychotherapists to offer VPT. Therefore, the perceived usefulness of VPT plays a critical role in the intention of both in-service psychotherapist and psychology students. Additionally, subjective norm (H2) and satisfaction with video conferencing (H4) were both positively related to the intention to offer VPT. These findings are consistent with previous studies that showed a positive relationship between these variables and intention in the context of e-learning (Liaw, 2008; Hussein, 2018). Social norm was previously shown to be an important predictor of behavior, particularly in novel situations (Hill et al., 1996). Because VPT is a novel experience for psychology students, the importance of subjective norm for intention was hypothesized. Interestingly, intention to offer was not significantly associated with either general self-efficacy (H5) or specific self-efficacy (H6) in our multiple regression. This lack of significance contrasts with the bivariate correlations observed between these variables and the intention to offer VPT, which demonstrated small to moderate relations. This suggests that self-efficacy loses its individual importance when included simultaneously with other variables in a multiple regression model. However, Aggelidis and Chatzoglou (2009) found that computer self-efficacy, along with other commonly used variables derived from the Technology Acceptance Model, made a significant contribution to explaining the variance in intention. Hence, further research is warranted to better understand the intricate interplay between (general and specific) self-efficacy, technology acceptance, and the intention to offer VPT, particularly in the context of varying sample characteristics. Lastly, we did not find any relation between study stage and the intention to offer VPT (H7). However, given that VPT is a relatively new subject with limited integration into university curricula, it is plausible that there are no significant differences in knowledge between undergraduate and graduate psychology students regarding VPT. Furthermore, Ajzen (1985) suggested that intention may vary depending on the temporal interval to actual behavior. As of the current system, psychology students in Germany require 3 to 5 years of training after graduation to become licensed practitioners, the long period until actual behavior may also explain why there was no significant difference in the level of intention to provide VPT between psychology students in bachelor’s and master’s programs. In summary, our data supports four out of the seven hypotheses, and the regression model demonstrated a remarkably high ability to explain the variance in the intention to offer VPT among individuals.

The results from the quantitative analysis have both theoretical and practical implications. The integration of variables from two distinct theoretical models, the Technology Acceptance Model and the Theory of Planned Behavior, in our study highlights the need for a tailored theoretical model specifically designed for the context of VPT among psychology students. The unique characteristics and considerations associated with VPT warrant the development of a comprehensive model that combines specific elements from existing models. By creating a hybrid model, researchers and practitioners can better capture the multifaceted nature of intention formation in the context of VPT. As shown by the present study, this model should incorporate factors such as attitudes, perceived usefulness, subjective norms, satisfaction with video conferencing, and potentially other relevant variables specific to VPT. It should also account for the potential interplay between these factors and their combined impact on the intention to offer VPT. For this purpose, the model of the present study could serve as a valuable starting point for future research, allowing for the exploration of additional variables and the examination of potential interactions and mediating factors that may further enhance our understanding of VPT intention formation.

In addition to these theoretical implications, the results from the multiple regression analysis also provide practical insights that hold relevance for educational institutions and universities being responsible for training psychotherapists. Promoting positive attitudes toward VPT among psychology students is essential for its successful integration into their practice. Educational institutions can play a pivotal role by incorporating coursework and training modules that create a conducive environment for exploring and understanding attitudes toward VPT and that highlight the advantages of VPT. These modules can involve discussions, case studies, and reflective exercises that allow students to analyze the formation of attitudes and engage in critical reflection on their own attitudes toward VPT. By providing opportunities for self-reflection and open dialogue, educational programs can effectively shape and cultivate positive attitudes toward VPT among psychology students. In addition to emphasizing the practical benefits and advantages of VPT, educational interventions can incorporate interactive methods to enhance students’ perception of its expected usefulness. Two effective approaches could involve incorporating role-playing exercises to provide firsthand experience and facilitating discussions that involve sharing and analyzing patient experiences. By engaging students in simulated VPT sessions, they can gain practical insights into the benefits and challenges associated with this mode of therapy. This immersive approach allows students to develop a deeper understanding of how VPT can effectively address psychological concerns. In addition, first-hand reports from patients who have benefited from VPT can provide valuable insight and real-life examples of the positive effects and outcomes of VPT. Through these interactive methods, educational institutions can effectively enhance students’ understanding and perception of the usefulness of VPT, thereby positively influencing their intention to adopt and provide it in their future practice. Recognizing the influence of social norms, educational programs should create an environment that stimulates a balanced reflection on the individual and societal meaning of VPT as well as on different expectations. Encouraging peer discussions and providing training on VPT within a social context can effectively reinforce normative beliefs and enhance students’ intention to offer VPT. By facilitating group discussions where students openly share their thoughts, experiences, and perspectives, educational programs can foster a supportive social context that cultivates a shared understanding of the significance of VPT. These peer discussions can serve as a platform for students to learn from one another, challenge existing norms, and collectively shape their intention to embrace VPT as a valuable therapeutic approach. Recognizing the relation of students’ past experiences with video conferencing on their intention to offer VPT, it is important to address any potential negative experiences and enhance general satisfaction with this mode of communication.

Educational institutions can implement two strategies to achieve this. Firstly, conducting best practice sessions for video conferencing (e.g., Rubinger et al., 2020) can provide students with practical guidance on optimizing their use of the technology. These sessions can cover topics such as optimizing audio and video quality, managing technical issues, and utilizing interactive features effectively. By equipping students with the necessary skills and knowledge, institutions can contribute to more positive experiences with video conferencing. Secondly, creating a space for open discussion and critical reflection on students’ past experiences with video conferencing can be beneficial. Facilitating group discussions in which students openly share their experiences, raise concerns, and analyze the reasons behind challenges encountered (such as technical issues or difficulties in mass conferences) can help identifying areas for improvement. This collaborative approach allows for the exploration of potential solutions and the development of strategies to address any barriers or concerns related to video conferencing. By considering these practical implications, institutions can better equip students with the knowledge and skills necessary to navigate the evolving landscape of mental health services.

### 4.2. Perceived advantages and disadvantages

Using a qualitative approach, we were able to identify major advantages and disadvantages of VPT perceived by psychology students (RQ1).

Our qualitative content analysis yielded two major categories of perceived advantages: firstly, easier access and delivery of psychotherapy (*low inhibition threshold, reduced mental and physical barriers, protection against disease and pandemic, accessibility, availability of the psychotherapist, lower costs, simplicity, anonymity, distance-related benefits* and *documentation capabilities*) and secondly, flexibility in psychotherapeutic care (*flexibility in terms of location, no travel necessary, psychotherapeutic care in rural areas, psychotherapeutic care worldwide, general flexibility and time-related benefits*). In addition to these two main categories, a small number of statements did not fit into any specific category and were assigned to a residual category. These statements may represent unique perspectives or additional benefits of VPT that were not captured by categories that required a minimum frequency of statements. Furthermore, a distinct category termed *spirit of the age* was identified, which encompassed statements related to the alignment of VPT with contemporary trends and societal expectations. It reflects the acknowledgment that VPT resonates with the current era and captures the evolving needs and expectations of individuals seeking mental health care.

When examining the results of each specific category in more detail, we observe that *low inhibition threshold* and *flexibility in terms of location* emerged as the two most frequently mentioned advantages of VPT. The category of *low inhibition threshold* encompasses the perceived ease and accessibility of initiating psychotherapy through VPT, removing barriers that may exist in traditional face-to-face settings. This includes the convenience of receiving therapy in the comfort of one’s own familiar environment, without the need to leave home. Additionally, it encompasses statements emphasizing that clients may feel more at ease and find it easier to open up during therapy sessions. Moreover, the category *flexibility in terms of location* highlights the convenience and accessibility of receiving psychotherapeutic care without the constraints of travel or geographical limitations. This includes the ability to continue therapy with the same therapist even after relocating and the freedom to choose a therapist regardless of location. Indeed, a meta-synthesis by McPherson et al. (2020) showed that patients reported improved flexibility and reduced barriers to access in VPT. In a study conducted by Eichenberg et al. (2021), patients in VPT reported a lower inhibition to discuss difficult topics. The authors also suggested that the physical distance between patients and therapists in VPT could be advantageous for individuals with attachment disorders and potentially improve the therapeutic relationship. Furthermore, in-service psychotherapists described the ability to address difficult issues more quickly, reduced shame and fewer inhibitions of patients, and increased focus on the therapeutic event (Eichenberg et al., 2021). Although *psychotherapeutic care in rural areas* and *worldwide* were not frequently mentioned by psychology students, it is noteworthy that these categories were rated as highly relevant. Notably, *psychotherapeutic care in rural areas* had the highest mean value in terms of relevance ratings among all advantage categories. Certain psychology students whose statements were classified under *flexibility in terms of location* may have also included rural or international flexibility, even if they did not explicitly state it. Consequently, this category may have been expanded at the expense of the two smaller categories. According to Donaghy et al. (2019), VPT has the potential to improve accessibility for individuals with limited mobility or mental illness, who may have trouble leaving their homes. Other advantages cited by psychology students are comparable with results of a qualitative study that asked in-service psychotherapists about benefits of VPT, such as better work-life balance, efficient use of time, the possibility of recordings, and no need for a therapy room (Buckman et al., 2021).

In terms of perceived disadvantages, we identified the following three major categories: firstly problems during psychotherapy sessions (*lack of closeness between patient and psychotherapist, lack of nonverbal cues, problems with technology or internet connection, therapeutic relationship suffers, no safe space for patients, communication problems and misunderstandings, more distraction possible, showing and recognizing empathy and emotions is difficult*), secondly limited application possibilities (*technology as a prerequisite, not appropriate for all disorders and patients* and *for all therapeutic methods, lower efficacy and less effectiveness, less opportunities for intervention*), and lastly other concerns (*lower commitment and motivation of patients, no benefits from leaving home, risk for data privacy, higher cognitive effort, and more difficult organization and bureaucracy*).

When examining the results of each specific category, the three most frequently mentioned disadvantages were *lack of closeness between patient and psychotherapist, lack of nonverbal cues,* and *problems with technology or internet connection*. Lack of nonverbal cues was also a perceived disadvantage of VPT by in-service psychotherapists (Eichenberg et al., 2021). A similar observation was made by Van der Vaart et al. (2014), who noted that VPT provides only a partial view of the other person, making it difficult for psychotherapists to respond to patients in the same way as they would during face-to-face sessions. Moreover, in-service psychotherapists described technological issues, poor internet connections and problems with online platforms as difficulties in providing treatment (Buckman et al., 2021). Patients also reported technical difficulties that disrupted VPT (Donaghy et al., 2019; McPherson et al., 2020). In addition to the technical difficulties that may arise during a VPT session, one of the disadvantages identified by psychology students is the requirement that patients have the necessary technical equipment and skills. This issue was found in other studies as well. Ghaneirad et al. (2021) reported that older age and lower levels of education were associated with lower use of VPT, which was also described by the students in the category *not appropriate for all disorders and patients*. Uscher-Pines et al. (2020) highlighted that the lack of internet access among underprivileged patients can lead to their exclusion from utilizing VPT. Furthermore, the found category *lower commitment and motivation of patients* is consistent with the results of a study by Eichenberg et al. (2021), in which in-service psychotherapists reported that patients who have difficulty establishing a stable therapeutic relationship are more likely to drop out of therapy. Lastly, psychology students fear a lack of a protected place and the lack of data security, which was also described as a barrier to VPT by McPherson et al. (2020).

Psychology students expressed many aspects that were consistent with those of practicing psychotherapists and patients regarding the advantages and disadvantages of VPT. In both the advantages and disadvantages, the students predominantly focused on aspects from the perspective of patients. Only a few categories specifically addressed advantages and disadvantages for therapeutic work (e.g., *lower costs, documentation capabilities, more difficult organization and bureaucracy*), indicating that the students have limited concrete knowledge about working as therapists and thus these aspects are less salient to them. Further research could be conducted by investigating individuals currently undergoing therapist training to gain a better understanding of their perspectives. This would shed light on which aspects they emphasize and whether the role of the therapist is given more prominence. Such insights could contribute to a more balanced understanding of the benefits and drawbacks of psychotherapy from both patient and therapist perspectives.

The results obtained from the qualitative content analysis also provide practical implications for the training of future psychotherapists. Firstly, the inclusion of VPT modules within the curriculum would provide specific instruction on the utilization of VPT in psychotherapy. This would encompass theoretical knowledge regarding effectiveness, ethical guidelines, and practical skills in navigating platforms. Secondly, practical exercises involving VPT could be incorporated, wherein students assume the roles of both therapist and patient within a virtual environment. Through this experiential approach, students would gain hands-on experience and become familiar with the challenges associated with VPT. Furthermore, the use of case studies and subsequent discussions could deepen understanding of specific VPT scenarios and facilitate analyses of the associated advantages and disadvantages. Group discussions would encourage the exploration of diverse perspectives and the development of various problem-solving approaches. To gain practical experience, students could engage in internships that involve the supervised application of VPT under the guidance of experienced therapists. Supervision sessions could specifically address the challenges and opportunities associated with VPT. Additionally, instructors could create an inclusive and open learning environment, where students are encouraged to engage in discussions and critically reflect upon the advantages and disadvantages of VPT in comparison to other therapeutic modalities. Encouraging dialogue and debate about different approaches will facilitate a comprehensive understanding of the strengths and limitations of each method. By promoting an atmosphere of open-mindedness and critical thinking, students can develop a balanced perspective on VPT and its role in psychotherapy. This approach will enable them to make informed decisions when considering the implementation of VPT in their future practice. A more detailed look at such learning opportunities is provided in the following section.

### 4.3. Desired learning opportunities

Our study identified desired learning opportunities that psychology students would like to have in their studies (RQ2). Here, we have identified two major categories of learning opportunities that psychology students desire in order to be equipped to offer VPT in their future practice. Firstly, various forms of practical training (*for technical skills, for conversational techniques, to build therapeutic relationships, to recognize and showing nonverbal cues* and *to increase motivation and compliance of patients, practical application as role-playing or self-experience and insights into the practice*), and secondly, diverse information on aspects of VPT (*information on (health insurance) legal requirements, on appropriate methods, on handling difficult situations during VPT sessions, on efficacy studies, on opportunities and limitations, on appropriate disorders and patients,* as well as *general information about VPT*).

Buckman et al. (2021) studied the perceptive of in-service psychotherapists and asked them what training and support they would need regarding VPT. Desired learning opportunities about research evidence for the effectiveness of VPT, training on the functions of the platforms used for VPT sessions and differences between platforms, as well as technical support and role-playing were mentioned among others. These findings are consistent with the results of our study, which focused on a younger and less experienced sample of psychology students. When examining the most frequently mentioned learning opportunities, we found that *training for technical skills* was the most mentioned category, followed by *practical application (role-playing/self-experience)*. It is noteworthy that the third most mentioned category was *general information about VPT*. This points to weaknesses in the current curriculum, in which little to no information about VPT is anchored. Consistent with this finding, we found no significant differences between psychology students enrolled in bachelor’s or master’s programs (RQ3) with regard to the number of statements made and the relevance ratings assigned to these statements. However, there was a slight variation in the order of the most frequently mentioned learning opportunities between the two groups. Specifically, the top five opportunities differed slightly, with bachelor’s students expressing a greater desire for additional training in conversational techniques via video conferencing, whereas master’s students more frequently expressed a desire for information on appropriate methods for VPT. The high relevance rating given by psychology students to all learning opportunities indicates their desire to acquire proper knowledge and skills for offering VPT.

We can derive practical implications for universities and training institutes from our findings regarding the desired learning opportunities. However, integrating these opportunities into the curriculum poses several challenges and difficulties. Nevertheless, the results of our study, as well as research showing the effectiveness of VPT (Backhaus et al., 2012; Hilty et al., 2013; Norwood et al., 2018; Berryhill et al., 2019a,b), highlight the importance of integrating VPT into the curriculum. Therefore, we propose a five-step plan: Firstly, as addressed in our study, it is important to determine whether VPT should be integrated at all. Our study provided an affirmative answer to this question, which is further supported by research demonstrating the effectiveness of VPT (Backhaus et al., 2012; Hilty et al., 2013; Norwood et al., 2018; Berryhill et al., 2019a,b). Secondly, universities and training institutes should assess the inclusion of these learning opportunities and examine the available capacity within the curriculum. This may involve considering the feasibility of incorporating a dedicated seminar on the topic or offering an optional supplementary course. Thirdly, it is crucial to determine the content to be delivered. Although our study has identified various desired learning opportunities, prioritization is necessary. Faculty members or trainers may hold different perspectives on priorities compared to the students in our sample. Fourthly, it is essential to explore the didactic implementation of the content and to identify any additional training needs for instructors in the field of computer-mediated communication. Moreover, the establishment of infrastructure is vital to facilitate targeted training. This includes ensuring reliable internet connections, providing an adequate number of high-quality computers, headsets, and microphones, and considering any other necessary resources to support effective implementation. Lastly, the fifth step involves conducting formative evaluation of the implemented program. This includes assessing its effectiveness, examining its impact on personal variables (pre-post), evaluating student satisfaction, and monitoring changes in intentions.

### 4.4. Limitations

The present study stands out due to its innovative focus, mixed-method design, and sample of psychology students. The findings have significant implications for the development of psychology study programs. However, some limitations should be noted.

First, the quantitative data in this study is limited to correlational and cross-sectional analyses. Therefore, the available data does not allow for any conclusive statements about the causal relationships between independent and dependent variables in the multiple regression model. However, this is a common approach in many studies that aim to explain the relative importance of several characteristics on behavioral intentions, as seen in previous studies (Balakrishnan, 2017; Rüth et al., 2022; Shahzalal and Adnan, 2022). In the future, it would be desirable to transform the study design to test for causality.

Second, the generalizability of the findings from this study to other countries and educational systems remains uncertain. The German system of psychotherapy training is distinct from others (Kaufmann et al., 2012), and it is likely that psychology students from different countries would consider different aspects of VPT than those in the present study. Moreover, legal requirements and healthcare insurance solutions for VPT vary across countries (*cf.* Kaufmann et al., 2012), which could influence participants’ responses. Nonetheless, given the widespread use of VPT during the pandemic, it is likely that VPT will continue to play a crucial role in psychotherapy training worldwide.

Third, it should be noted that this study only assessed psychology students’ intention to use VPT after graduation, which can take several years. The continuous development of their intention over time was not examined in this study. While intention is considered a key predictor of behavioral performance according to Fishbein and Ajzen (1975), it is not a guarantee. Whether these psychology students will actually offer VPT when they become licensed psychotherapists cannot be determined from the current results and requires further investigation. A longitudinal study could provide valuable insights into this question.

Fourth and last, it should be noted that the study ran during the COVID-19 pandemic, so responses could have been influenced by this extreme situation. For example, it can be assumed that the category *protection against disease and pandemic* would have been smaller, and that the keyword “pandemic” would not have been mentioned at all. The focus of the participants could have been on a different topic under different circumstances. In addition, results from Doran and Lawson (2021) indicated that attitudes toward VPT have become significantly more positive during the pandemic, as it was perceived as important, necessary, and effective during the pandemic. Therefore, it is important to examine whether these results will persist beyond the acute phase of the pandemic. It is possible, however, that the relevance of VPT will continue as the next generation of psychotherapists realize that digital mental health care continues to be of great importance.

## 5. Conclusion

Overall, this mixed-method study provided insights into psychology students’ intention to offer VPT after their graduation, their perceived advantages and disadvantages of VPT, as well as desired learning opportunities for study programs. The multiple regression model with intention to offer VPT as dependent variable and attitudes toward VPT, subjective norm, satisfaction with video conferencing, general self-efficacy, self-efficacy in video conferencing, and current study stage as independent variables explained 73% of variance between participants. Our results indicate that promoting positive attitudes toward VPT among psychology students is essential for successful integration into practice. Educational institutions should incorporate coursework and training modules that foster exploration and understanding of VPT attitudes, highlighting its advantages. Interactive methods like role-playing and patient experience discussions can enhance perception of VPT’s usefulness. These strategies could cultivate a supportive mindset among future psychotherapists. Furthermore, the psychology students showed a generally high intention to offer VPT after graduation. The high intention of psychology students to offer VPT after graduation is an important finding that has implications for various stakeholders, including health insurers. The decision on whether VPT will have a long-lasting future after the pandemic largely depends on health insurers, who play a crucial role in determining whether VPT sessions are reimbursable or not. If VPT is not considered a viable option for psychotherapy by health insurers, it may limit the accessibility of mental healthcare services for many individuals. Therefore, it is important for health insurers to take into account the results of this study and consider the potential benefits of VPT in making decisions about reimbursement. Furthermore, our study offers a valuable insight into the specific learning preferences of psychology students regarding VPT. These preferences should be taken into consideration by universities and training institutions when designing effective teaching methods. To address this, we suggest five-step plan tailored to the specific needs of psychology students. This plan can serve as a practical framework for universities and training institutions to effectively incorporate VPT-related content into their curricula, ensuring an optimal learning experience for future psychotherapists. However, our research only captures the opinions of future psychotherapists. Further research is needed to examine the extent to which the perceived advantages and disadvantages align with the reality of VPT and how VPT can be successfully integrated alongside traditional psychotherapy.

## Data availability statement

The raw data supporting the conclusions of this article will be made available by the authors, without undue reservation.

## Ethics statement

Ethical review and approval was not required for the study on human participants in accordance with the local legislation and institutional requirements. The patients/participants provided their written informed consent to participate in this study.

## Author contributions

JM, JN, and KK developed the study idea, designed the study, interpreted the results, and wrote the manuscript. JN collected the data. JM and KK organized and supervised data collection. JM and JN performed the analyses. KK supervised the analyses. All authors contributed to the article and approved the submitted version.
