# Supplementary material for: Understanding psychology students’ perspective on video psychotherapy and their intention to offer it after graduation: a mixed-methods study
Source: Front Psychol. 2023 Oct 19;14:1234167. doi: 10.3389/fpsyg.2023.1234167 (PMC10620503; doi:10.3389/fpsyg.2023.1234167)
Supplement: Supplementary file 1 [file Data_Sheet_1.pdf]

# *Supplementary material*

**Table S1.** Detailed description of the categories for perceived advantages of video psychotherapy.

| Category | Definition of the category |
|---|---|
| 1. Low inhibition threshold | This category includes advantages of VPT that address the low inhibition threshold for patients to participate in VPT or to start psychotherapy in general. This includes aspects that describe that the start of psychotherapy is perceived as easier due to the possibility of VPT. In addition, statements that describe the possibility of patients being able to remain at home in their familiar environment and not having to leave the house for psychotherapy belong in this category. This category also includes aspects that describe how patients feel more comfortable in a familiar environment and therefore can be more open to the psychotherapist or have fewer inhibitions about opening up. Statements about the potential advantages of being physically distant from the therapist belong in this category. |
| 2. Flexibility in terms of location | This category includes advantages of VPT that address flexibility in terms of location. This includes the advantage of not having to change psychotherapists after a move and the possibility of choosing a suitable psychotherapist regardless of one's place of living. Statements describing that psychotherapy can be continued during a vacation belong to this category as well. |
| 3. No travel necessary | This category includes the advantages of VPT resulting from the elimination of travel distances. More general statements, describing that the patient (or even the psychotherapist) does not need to drive to the office, fall into this category. This also includes statements that explicitly describe lower costs, time savings, and environmental protection by minimizing travel distances. |
| 4. General flexibility | This category includes advantages of VPT that describe a general and undifferentiated flexibility. Further, this includes statements about the possibility of individual coordination with patients, e.g., that cancellation of appointments can be prevented by video psychotherapy. In addition, the flexibility for the psychotherapists belongs to this category, e.g., the possibility to work from home or to apply new methods with the help of technology. |
| 5. Reduced mental and physical barriers | This category includes advantages of VPT that address reduced barriers, i.e., that VPT is well suited for some disorders or patients. This includes statements about reduced barriers for physically and mentally affected people who cannot or do not want to leave their homes. Also, statements addressing the possibility of mental health treatment via VPT for anxious people or parents with children who have difficulties leaving the house as a result, belong in this category. It is important to note that VPT must be mentioned as an advantage for certain groups of people. |
| 6. Protection against disease and pandemic | This category includes advantages of VPT that relate to psychotherapeutic care during illness or the (COVID-19) pandemic. It includes statements about protection from infection (including COVID-19) and participation in VPT from home despite mild illness. Particularly considering the pandemic, statements that describe the protection of at-risk groups from COVID-19 infection belong to this category. |
| 7. Accessibility | This category includes advantages of VPT that address the accessibility of VPT. This includes statements about the increased patient capacity of psychotherapists, the possibility of offering more psychotherapy appointments and correspondingly shorter waiting times for a psychotherapy appointment for patients, and improved patient care. Thus, aspects that describe patients being able to get an appointment for psychotherapy more easily belong to this category. |
| 8. Time-related benefits | This category includes advantages of VPT that address the time-related benefits of VPT. This includes statements about flexible appointment scheduling and easier integrating of VPT into daily life. Also, statements that describe VPT as offering the opportunity to save time belong in this category. Time savings for the specific reason of reduced travel do not belong in this category, but in category 3. |
| 9. Availability of the psychotherapist | This category includes advantages of VPT that target the availability of psychotherapists. This includes all aspects of availability that are addressed, such as easier, better, or faster availability due to the possibility of using VPT, and statements describing opportunities for patients and psychotherapists to quickly update each other. Additionally, statements about availability during emergencies belong to this category. |

| | |
|---|---|
| 10. Lower costs | This category includes advantages of VPT that address the costs saved by VPT. This includes, for example, statements about reduced costs due to the reduction/elimination of room rent for psychotherapists but also cost savings in general. It is important to note that statements on reduced costs due to reduced travel do not belong in this category, but in category 3. |
| 11. Simplicity | This category includes advantages of VPT that relate to the simplicity of VPT. This includes aspects that describe the ease of use and reduced effort. In addition, this category includes statements that refer to the uncomplicated nature of VPT. |
| 12. Psychotherapeutic care in rural areas | This category includes advantages of VPT that address enabling rural and isolated regions to receive mental health care via VPT. This includes statements describing that VPT can provide care to regions where it is difficult to obtain psychotherapy treatment. |
| 13. Psychotherapeutic care worldwide | This category includes advantages of VPT related to the possibility of receiving psychotherapeutic care through VPT on an international level. This includes care during a time abroad, e.g., during a vacation abroad, an internship abroad, a job abroad, etc. Statements describing that psychotherapy can take place in one's native language even if the patients are not in the same country as the psychotherapist also belong to this category. |
| 14. Anonymity | This category includes advantages of VPT that address the positive aspects of anonymity. This includes statements about the reduced stigmatization of patients and the positively perceived anonymity due to the reduction of face-to-face interaction with other patients in the psychotherapist's office waiting room. |
| 15. Spirit of the age | This category includes all advantages of VPT that describe VPT as the spirit of the age, modern, and state-of-the-art. Statements describing VPT as a step towards modernizing the health care system belong to this category. In addition, this category includes statements describing, that patients might want VPT, and that the psychotherapist can meet their desire. |
| 16. Documentation capabilities | This category includes all advantages of VPT that pertain to the documentation capabilities of VPT. This includes statements describing, that sessions can be recorded for supervision, as well as video recordings can be reviewed and analyzed, or that psychotherapy is made more auditable. |
| 17. Distance-related benefits | This category includes advantages of VPT that relate to the interpersonal distance between psychotherapist and patient. Here it is explicitly not about the local distance, but about the interpersonal distance due to VPT. These include advantages such as the possibility of maintaining a professional distance via VPT. Otherwise, the statements would belong in another category depending on their key message. |
| 18. Residual category | This category includes statements that cannot be assigned to the defined categories, are not understood by the raters, or do not make sense in terms of content related to the question. |

**Table S2.** Detailed description of the categories for perceived disadvantages of video psychotherapy.

| Name of the category | Definition of the category |
|---|---|
| 1. Lack of closeness between patient and psychotherapist | This category includes disadvantages of VPT that concern the (spatial) distance between patient and psychotherapist. This includes descriptions of the feeling of lack of personal closeness. Statements describing that anonymity is intensified by the distance and that there is a virtual barrier between the participants of the VPT belong to this category. In addition, statements that describe the absence of atmosphere, energy, and the therapeutic spirit that arises during in-person psychotherapy belong to this category. |
| 2. Lack of nonverbal cues | This category includes disadvantages of VPT that address limited or missing nonverbal communication aspects via video. This includes statements addressing the limited field of vision due to the screen, less visible body language, as well as facial expressions and gestures. However, statements about the difficulty of reading nonverbal cues and the lack of eye contact belong to this category. |
| 3. Problems with technology or internet connection | This category includes disadvantages of VPT, addressing connection problems during the VPT session that are caused by technical problems and internet breakdowns. This includes statements that describe how these problems can harm the flow of conversation. It is important that terms such as “internet/technology disruption” or “internet/technology problems” be present in the key statement. |
| 4. Therapeutic relationship suffers | This category includes disadvantages of VPT that address the reduced therapeutic relationship or describe that the therapeutic relationship between therapist and patient is more difficult to establish via VPT (e.g., due to spatial distance or video conferencing). This also includes aspects that describe that building trust in VPT is more difficult. The focus of the statements is on the interpersonal relationship. |
| 5. Technology as a prerequisite | This category includes disadvantages of VPT that address the problem that technical devices and the internet are preconditions for VPT (for patients and/or psychotherapists). This includes statements describing that media literacy, the technology itself, the internet, the costs due to technology, etc. must be available for the use of VPT to be possible. This is explicitly not about problems with the internet or with technology (during a VPT session). Indeed, such statements belong in category 3. |
| 6. No safe space for patients | This category includes disadvantages of VPT that address the problem of patients being in their own homes and thus not having a safe space or protected privacy. This includes statements that patients do not have a safe psychotherapy setting and remain in their potentially problematic environment. Descriptions of consequences, such as inhibitions to open, and talking about intimate content, also belong in this category. Importantly, the statements that belong to this category focus on the “lack of safe space” and “privacy”, which in turn causes disadvantages for VPT. |
| 7. Not appropriate for all disorders and patients | This category includes disadvantages of VPT that address the problem that VPT is not suitable or possible for all disorders and patients. Included are all statements regarding exclusions of certain individuals from VPT, e.g., due to associated costs, lack of technical competence, etc. It is important that the focus of the core statement is on the exclusion of individuals or disorders. |
| 8. Not appropriate for all therapeutic methods | This category includes disadvantages of VPT that relate to the problem that not all therapeutic methods can be performed via VPT. This includes statements about specific methods that are less implementable, such as (counter-)transference processes, group sessions, working together on worksheets, etc. |
| 9. Communication problems and misunderstandings possible | This category includes disadvantages of VPT that address the disrupted communication due to the video format. This includes statements of disturbed speech flow, delayed interaction, loss of information by the video conferencing, but also a reduced depth of conversation and a worsened exchange. Descriptions about the lack of face-to-face communication or a higher likelihood of misunderstandings also belong in this category. It is not about problems caused by the internet or technology. Such statements belong in category 3. |
| 10. Lower commitment and motivation of patients | This category includes disadvantages of VPT that address the problem that VPT is perceived by patients as less obligatory. This also includes statements about psychotherapy interruptions during VPT sessions or during the process of psychotherapy by the client. Statements concerning a lower motivation of patients (compared to face-to-face psychotherapy in presence) belong to this category. |

| | |
|---|---|
| 11. More distraction possible | This category includes disadvantages of VPT that address the easier distraction in general or by the home environment. This includes statements describing distractions and interference from family, technical devices, as well as seeing oneself on the screen, etc. |
| 12. No benefits from leaving home | This category includes disadvantages of VPT, that address the problem that the benefits of leaving the house (as they would occur in face-to-face psychotherapy) are absent in the case of VPT. This includes statements describing the lack of traveling to the office that can be used to prepare for or summarize the psychotherapy session, increasing loneliness, staying in one's comfort zone, and not having the rituals around the psychotherapy visit. |
| 13. Showing and recognizing empathy and emotions is difficult | This category includes the disadvantages of VPT, which address the difficulty of showing, feeling, and recognizing empathy and emotion or responding to the patient's feelings due to video conferencing. Statements referring to the difficulty of perceiving or showing nonverbal cues via video conferencing do not belong to this category, but to category 2. |
| 14. Lower efficacy and less effectiveness | This category includes disadvantages of VPT that address lower effectiveness or efficiency of VPT compared to face-to-face psychotherapy in presence. This includes statements about less influence of VPT on patients, as well as aspects that are described as being inferior in comparison to face-to-face psychotherapy in presence. In addition, descriptions that patients can be assessed worse by the psychotherapists fit into this category. Statements that less serious VPT offerings may emerge also belong in this category. |
| 15. Risk for data privacy | This category includes disadvantages of VPT that concern the (questionable) data security regarding VPT. This also includes statements describing the possibility that unauthorized recordings of the psychotherapy session can be made. |
| 16. Less opportunities for intervention | This category includes disadvantages of VPT that address the psychotherapists' lack of opportunities for intervention and interaction during VPT sessions. This also includes statements about limited possibilities of intervention in crisis situations of the patients. |
| 17. Higher cognitive effort | This category includes disadvantages of VPT that relate to the increased effort due to the video format during VPT. This includes aspects such as viewing a screen for a longer period, the resulting eye stress, maintaining concentration, and the general high level of cognitive effort during VPT. |
| 18. More difficult organization and bureaucracy | This category includes the disadvantages of VPT that address the (additional) organization and bureaucracy to offer VPT sessions to be more complicated and difficult for psychotherapists. This includes statements describing a more difficult implementation of the VPT, the additional digitalization of the procedures, the lack of transparency in the health insurance companies' payment, etc. |
| 19. Residual category | This category includes statements that cannot be assigned to the defined categories, are not understood by the raters, or do not make sense in terms of content related to the question. |

**Table S3.** Detailed description of the categories for desired learning opportunities for video psychotherapy in psychology studies.

| Name of the category | Definition of the category |
|---|---|
| 1. Training for technical skills | This category includes desired learning opportunities for VPT that address technical skills, e.g., hardware and software skills. This also includes statements that describe the desire of learning about appropriate programs and hardware, as well as practicing their use or acquiring technology-related skills. |
| 2. Practical application (role-playing/self-experience) | This category includes desired learning opportunities that address the practical application of VPT. This includes desires for role-playing and self-experience to simulate and learn about VPT in a practical way. |
| 3. General information about VPT | This category includes desired learning opportunities for the VPT that address general information about the VPT. This includes statements that address how general information should be delivered in study (e.g., lecture, seminar). In addition, statements that desire looking at differences between face-to-face and VPT or specifics of VPT fall into this category. Desired learning opportunities on how to set up and organize a VPT session also belong in this category. |
| 4. Training for conversational techniques via video conferencing | This category includes desired learning opportunities for VPT that address conducting conversations via video conferencing. This includes desires for information on differences to conversation techniques in face-to-face communication and the implementation of appropriate exercises. Statements describing the desire to learn about maintaining the flow of conversation and communication features via video are also included in this category. |
| 5. Information on (health insurance) legal requirements | This category includes desired learning opportunities for VPT that address information on (health insurance) legal issues, such as privacy or data protection, legal specifics, and health insurance legal accounting for VPT. |
| 6. Training to build therapeutic relationship via video conferencing | This category includes desired learning opportunities for the VPT that address building a therapeutic relationship via video conferencing. This includes statements describing the desire to learn how to establish a therapeutic relationship despite physical distance, as well as aspects of showing empathy and encouragement or recognizing feelings about video conferencing. In addition, statements about learning how to achieve personal closeness belong in this category. |
| 7. Information on appropriate methods for VPT | This category includes all desired learning opportunities that address the specific methods of VPT. This includes learning opportunities on methods that may or may not be feasible. |
| 8. Insights into the practice of VPT | This category includes desired learning opportunities that focus on insights into the practice of VPT. This includes desires to learn with experiential reports from and conversations with video psychotherapists and patients, as well as watching sample videos of VPT. |
| 9. Information on handling difficult situations during VPT sessions | This category includes desired learning opportunities for the VPT that address information about difficult situations that occur, for example, due to physical distance. This includes statements about how to deal with a patient's dissociation, a patient's cancellation of the psychotherapy session, or disconnection due to technical problems. Requests for methods how to deal with difficult situations also belong in this category. |
| 10. Information on efficacy studies on VPT | This category includes desired learning opportunities on VPT that address information about the effectiveness of VPT and related effectiveness studies or the current state of studies on VPT. This also involves studies on methodological aspects of VPT. It is important that the statements have the desire for scientific information as their focus. |
| 11. Information on opportunities and limitations of VPT | This category includes desired learning opportunities about VPT that address information about advantages and disadvantages or opportunities and limitations of VPT. This also includes advantages and disadvantages or opportunities and limitations of VPT that may occur because of video conferencing. Keywords such as "advantages", "disadvantages", "limitations", "opportunities" or similar should be present in the statements. |

| | |
|---|---|
| 12. Training to recognize and showing nonverbal cues via video conferencing | This category includes desired learning opportunities that address nonverbal cues via VPT. The the desire to learn how to use nonverbal cues such as facial expressions, body language, and gestures, as well as how to recognize them in the other person belong in this category. |
| 13. Information on appropriate disorders and patients | This category includes desired learning opportunities for VPT that address information about disorders or patients appropriate for VPT. This includes statements related to the question for which disorders/patients VPT is more appropriate and for which or whom it is less appropriate. |
| 14. Training to increase motivation and compliance of patients | This category includes all desired learning opportunities that address creating or increasing client motivation and compliance. |
| 15. Residual category | This category includes statements that cannot be assigned to the defined categories, are not understood by the raters, or do not make sense in terms of content related to the question. |
